# Compensatory responses can alter the form of the biodiversity–function relation curve

**DOI:** 10.1098/rspb.2019.0287

**Published:** 2019-04-17

**Authors:** Matthias S. Thomsen, Jasmin A. Godbold, Clement Garcia, Stefan G. Bolam, Ruth Parker, Martin Solan

**Affiliations:** 1 Ocean and Earth Science, National Oceanography Centre Southampton, University of Southampton, Waterfront Campus, European Way, Southampton SO14 3ZH, UK; 2 Biological Sciences, University of Southampton, Highfield Campus, Southampton SO17 1BJ, UK; 3 Centre for Environment, Fisheries and Aquaculture Science (Cefas), Pakefield Road, Lowestoft, Suffolk NR33 0HT, UK

**Keywords:** evenness, ecosystem function, effect traits, response traits, extinction debt, species response

## Abstract

There is now strong evidence that ecosystem properties are influenced by alterations in biodiversity. The consensus that has emerged from over two decades of research is that the form of the biodiversity–functioning relationship follows a saturating curve. However, the foundation from which these conclusions are drawn mostly stems from empirical investigations that have not accounted for post-extinction changes in community composition and structure, or how surviving species respond to new circumstances and modify their contribution to functioning. Here, we use marine sediment-dwelling invertebrate communities to experimentally assess whether post-extinction compensatory mechanisms (simulated by increasing species biomass) have the potential to alter biodiversity–ecosystem function relations. Consistent with recent numerical simulations, we find that the form of the biodiversity–function curve is dependent on whether or not compensatory responses are present, the cause and extent of extinction, and species density. When species losses are combined with the compensatory responses of surviving species, both community composition, dominance structure, and the pool and relative expression of functionally important traits change and affect species interactions and behaviour. These observations emphasize the importance of post-extinction community composition in determining the stability of ecosystem functioning following extinction. Our results caution against the use of the generalized biodiversity–function curve when generating probabilistic estimates of post-extinction ecosystem properties for practical application.

## 1. Introduction

Populations can respond to the loss of, or reduction in, the number of individuals or species in a community through various compensatory mechanisms, including numeric [1–3], biomass [4,5] and/or *per capita* processing rate responses [6], or via mechanisms that effectively absorb disturbances through changes in trophic interactions [7]. Such expressions are often associated with adjustments in the competitive balance between species [8–10], contributing to resource-release and new opportunities [7,11–14] or a change in the prevalence and strength of functionally important species interactions [15] that allow a subset of species to prosper and exhibit compensatory responses to novel circumstances. Intuitively, such fundamental changes in community structure are likely to modify community contributions to functioning and, ultimately, define the long-term legacy of a perturbation. Yet, the effects of compensatory responses on ecosystem functioning are not well understood, despite recognition that there are multiple instances of species compensation in geological records following major perturbation events [16–19], some of which appear to be a part of a global pattern [20]. Many of these events are associated with regime shifts, in which substantive rearrangement in functional trait composition and the use of novel space following species decline takes place and has concomitant effects on ecosystem properties [20]. These effects are not necessarily negative, the realized level of functioning can be conditional on trophic structure and/or the variation within, and covariation between, the response and effect traits of the surviving community [21]. This means that the level of functioning may be maintained, reduced or enhanced relative to the pre-extinction condition. It follows, therefore, that the general form of the positive but decelerating biodiversity–ecosystem functioning curve that emerges from 2 decades of experimentation [22,23] is unrepresentative of the most likely post-disturbance outcome for ecosystem functioning. Many community processes and dynamics that are known to have compensatory attributes [24,25] have not been fully considered within the biodiversity–function experimental framework.

Recent studies have shown that the order in which species are lost can influence ecosystem properties [25–27], and that the potential of the surviving community to compensate for the loss or reduction in functionally important species will be dependent on the level of functional redundancy [28–31] and on realized levels of species richness [32–35]. Evidence suggests that the effects of compensation can increase (over-compensation, [5,36,37]), maintain (complete compensation [1]) or reduce (partial to no compensation [38]) ecosystem functioning, and that the ecosystem consequences of biodiversity loss could be buffered by the presence of a low number of functionally important species [5,39]. While this may be appealing from a management or conservation perspective, such a synthesis ignores other important aspects of post-perturbation community dynamics. In particular, recent numeric simulations [26,27] and field observations [5] suggest that ecosystem responses to perturbation may be dependent on the type of compensation that develops in the surviving community [25], but because these conclusions can take extended time periods to develop [5], they have not been empirically tested and are yet to be incorporated into ecosystem models.

Here, we experimentally explore how the effects of biomass compensation following the loss of sediment-dwelling marine invertebrates may affect sediment mixing and associated levels of nutrient generation in model benthic communities. Specifically, invertebrate communities were assembled to reflect a sequence of species loss that was random or ordered by body size or rarity to represent likely generic sources of extinction risk [40–43]. We simulated post-extinction compensation by introducing additional individuals of the ‘surviving’ species, circumventing the need for lengthy studies that incorporate recruitment and growth over several generations. It was anticipated that functional compensation would be less pronounced in communities in which extinction order is related to body size, as body size is often correlated with benthic ecosystem functioning [26] and is considered a key species trait at the population level [44]. Similarly, given that species which occur at low abundances generally contribute little to the ecosystem function inventory, compensation in response to the loss of rare species was expected to lead to elevated levels of functioning (*sensu*, the insurance hypothesis [45,46]). Irrespective of extinction scenario, we anticipated that compensatory effects would be more accentuated in communities of high evenness, because traits are more evenly distributed relative to those communities assembled to reflect natural evenness levels where functional dominance is prevalent.

## 2. Methods

### (a) Faunal and sediment collection

Sediment (mean ± s.e., *n* = 4: *D*_50_ = 6.122 ± 0.105 µm; total organic carbon = 11.058 ± 1.087%) and specimens of the gastropod *Peringia ulvae* were collected from the Hamble Estuary, UK (50°52′22.8″ N 1°18′48.9″ W), while the amphipod *Corophium volutator* was collected from Hayling Island, UK (50°49′56.9″ N 0°58'36.8″ W) in April 2015. Both *P. ulvae* and *C. volutator* were collected by sieving surface sediment (1 mm and 500 µm mesh, respectively). Individuals of the polychaete *Hediste diversicolor* were collected by hand from Langstone Harbour, Portsmouth (50°50′46.5″ N 1°00′05.3″ W). These three species co-occur at the two sites, but sampling location reflected logistical convenience. The sediment was sieved (500 µm mesh) in a seawater bath to remove macrofauna, allowed to settle (48 h to retain the fine fraction, less than 63 µm), and homogenized by stirring prior to distribution between experimental aquaria.

### (b) Experimental design

**Figure 1.**
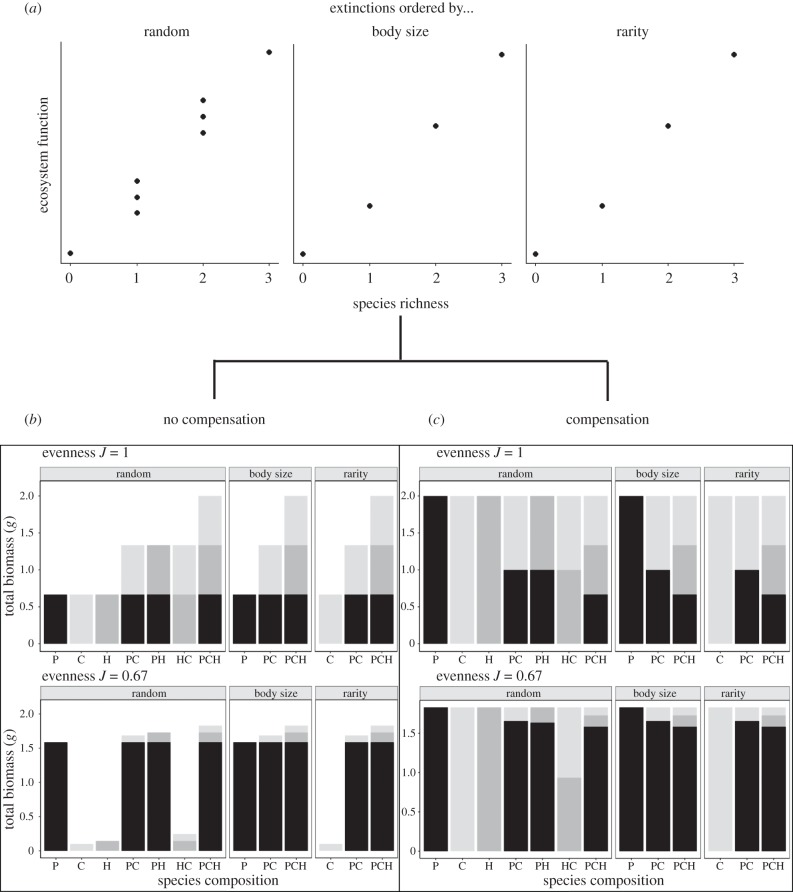
Summary of the experimental design. Communities were assembled to reflect extinction scenarios that assumed (*a*) random extinction, representing the full spectrum of possible species combinations, versus trait-based extinctions ordered by body size (i.e. body mass) or relative abundance (rarity). Each scenario of extinction consisted of (*b*) a set of non-interactive communities that experienced no biomass compensation versus (*c*) a set of interactive communities that experienced complete biomass compensation in response to declining species richness. The design was repeated across two levels of evenness that represent those typical of experimental (*J*^1^) versus natural (*J*^0.67^) systems. C, *Corophium volutator*; H, *Hediste diversicolor*; P, *Peringia ulvae* for monoculture and combination of abbreviations for species mixtures. Shading of bars indicates the proportional representation of each species (C, light grey; H, dark grey; P, black).

We assembled replicate (*n* = 4) transparent acrylic aquaria (12 × 12 cm, 35 cm high; 10 cm sediment overlain by 20 cm seawater, salinity approx. 33) containing all possible permutations of species composition (no macro-invertebrates, species in monoculture and in combinations of two or three species) for three scenarios of species loss, where the probability of extinction was either random (1/*n*, eight assemblages) or ordered sequentially in proportion to body size (largest species expire first, four assemblages) or relative abundance (rarity, species with lowest abundance expire first; four assemblages). To test the effects of post-extinction biomass compensation, this set of aquaria were duplicated in order to include a set of non-interactive communities that experienced no biomass compensation, i.e. species biomass declined with loss of species; versus a set of interactive communities in which complete biomass compensation was simulated by maintaining the total biomass of each community across the remaining species (electronic supplementary material, tables S1 and S2). This design was repeated across two levels of evenness that represent organism density distributions that are either evenly distributed and typical of most biodiversity–ecosystem function experiments (*J*^1^) or that contain a dominance hierarchy more typical of a natural system (*J*^0.67^, based on field observations; [47–49]). Hence, the experimental design required a total of 256 aquaria (figure 1), all of which were maintained in a water bath at 12°C under a 12 L : 12 D light : dark regime and continually aerated for 12 days.

### (c) Measurements of ecosystem process and functioning

Fluorescent sediment profile imaging (f-SPI) was used to quantify the extent of infaunal particle reworking [50]. This technique allows the redistribution of an optically distinct particulate tracer (60 g red coloured sand aquaria^−1^, fluorescent under ultraviolet light; Brianclegg Ltd, UK) to be quantified from a composite image (Canon 400D, set to 10 s exposure, aperture f5.6 and ISO400; 3888 × 2592 pixel, effective resolution 62.5 µm pixel^−1^) of the four sides of each aquarium using a custom-made, semi-automated macro within ImageJ (1.47v). From these data, the mean (^f-SPI^*L*_mean_, a time-dependent indication of mixing), median (^f-SPI^*L*_med_, the extent of mixing typically encountered over the short term) and maximum (^f-SPI^*L*_max_, the extent of mixing, including infrequent deep mixing events, achieved over the long term) depths of particle redistribution were calculated as an indicator of macro-invertebrate reworking [51]. In addition, surface boundary roughness (SBR, the maximal vertical deviation of the sediment–water interface) was determined as an indication of surficial activity.

Burrow ventilation, a significant transport mechanism in the exchange of solutes between the pore water and overlying water, was estimated on day 12 from changes in the concentration of the inert tracer sodium bromide (Δ[Br^−^], mg l^−1^; [52] over a 4 h period (aeration disabled) following the addition of sodium bromide (2.74 g, raising water column concentration of bromide to 9.25 mmol l^−1^), and quantified using a Tecator flow injection auto-analyser (FIA Star 5010 series).

Water column nutrient concentrations ([NH_4_–N], [NO*_x_*–N] and [PO_4_–P]) were determined (Tecator flow injection auto-analyser, FIA Star 5010 series) from samples (10 ml, 0.45 µm pre-filtered, day 12) taken from the centre of each aquarium approximately 5 cm above the sediment–water interface.

### (d) Statistical analyses

A total of seven statistical models were developed, one for each of the dependent variables (SBR, ^f-SPI^*L*_mean_, ^f-SPI^*L*_med_, ^f-SPI^*L*_max_, Δ[Br^−^], [NH_4_–N], [NO*_x_*–N], [PO_4_–P]) with species richness, extinction order and compensation as fixed effects. The control treatments were excluded from the statistical analyses, as the focus is to assess the effects of different extinction scenarios and not the presence/absence of macrofauna on ecosystem properties. The initial linear models were assessed visually for normality (*Q*–*Q* plot), homogeneity of variance (plotted residual versus fitted values) as well as for influential data points (Cook's distance) [53]. In cases where data exploration indicated heterogeneity of variances, relationships were defined using restricted maximum likelihood and generalized least-squares (GLS) estimation [54]. The use of GLS allows the variance structure to be modelled using appropriate variance functions (here ‘varIdent’ for nominal explanatory variables) rather than transforming the data [53,54]. The model with and without the variance covariate term was compared using Akaike information criterion (AIC, model improvement indicated by a reduction of greater than or equal to 2 units) and by visual inspection, plotting residuals versus fitted values, in order to identify the optimal random effects structure for each response variable [53,55]. The optimal fixed effects model was estimated using maximum-likelihood (ML) estimation and determined using a backward selection procedure informed by AIC [55]. All statistical analysis was performed using the ‘R’ statistical and programming environment [56] and the ‘nlme’ R package (v. 3.1-128, 2016) [57].

## 3. Results

**Figure 2.**
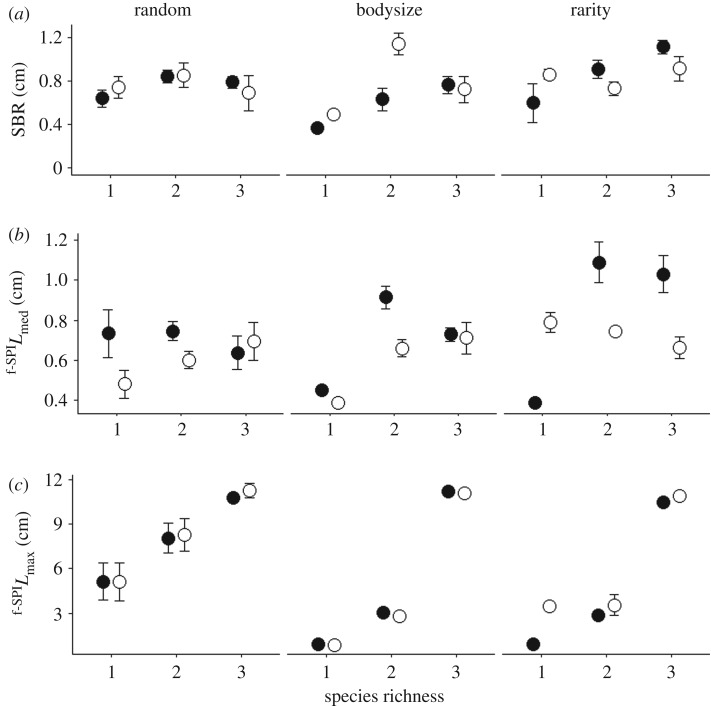
Interactive effects of extinction scenario (random or ordered by body size or rarity), species richness and biomass compensation (present, closed circle; absent, open circle) on mean (±s.e., *n* = 4) (*a*) SBR, (*b*) median depth of particle reworking (^f-SPI^*L*_med_), and (*c*) maximum depth of particle reworking (^f-SPI^*L*_max_) for communities with even species distribution (*J*^1^). For visual clarity, compensation levels are horizontally offset.

**Figure 3.**
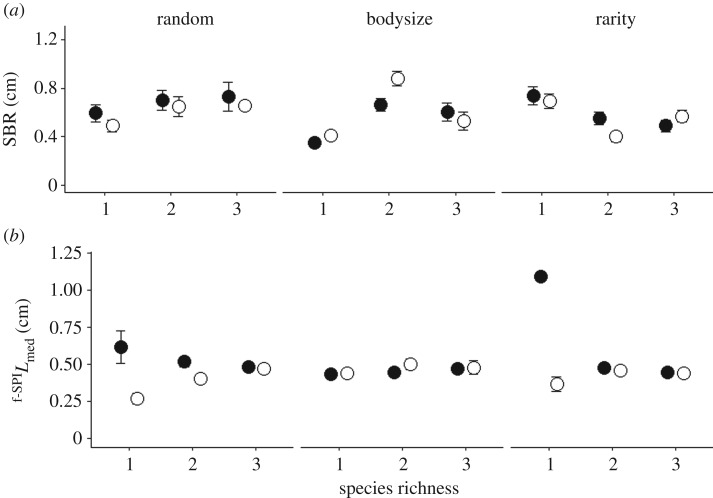
Interactive effects of extinction scenario (random or ordered by body size or rarity), species richness and biomass compensation (absent, open circle; present, closed circle) on mean (±s.e., *n* = 4) (*a*) SBR and (*b*) maximum depth of particle reworking (^f-SPI^*L*_max_) in uneven communities (*J*^0.67^). For clarity, compensation levels are offset horizontally.

**Figure 4.**
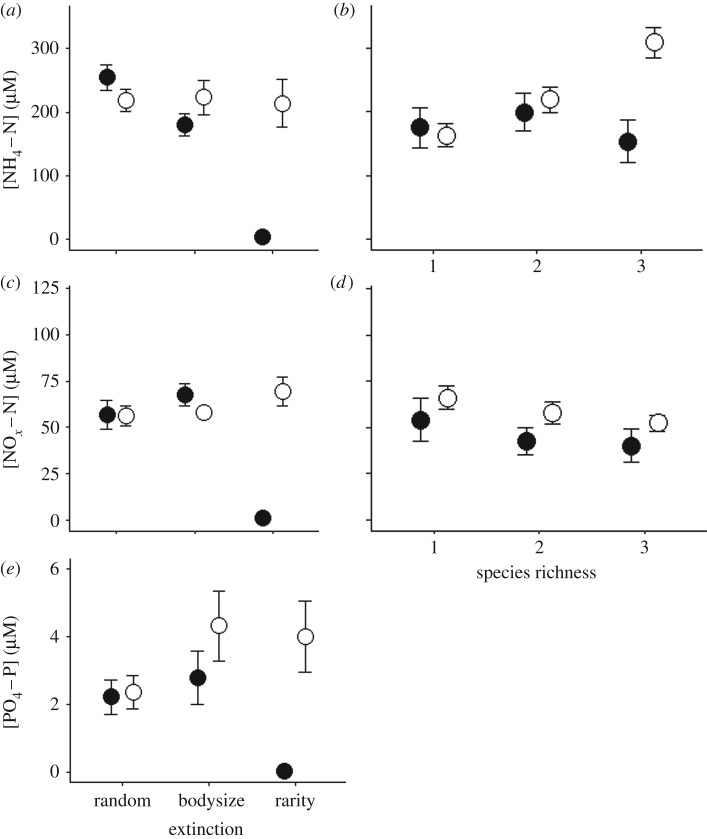
Effects of (*a*,*c*,*e*) extinction scenario (random, body size, rarity) or (*b*,*d*) species richness in the absence (open circle) versus presence (closed circle) of compensation in even (*J*^1^) communities on mean (±s.e., *n* = 4) [NH_4_–N], [NO*_x_*–N] and [PO_4_–P].

**Figure 5.**
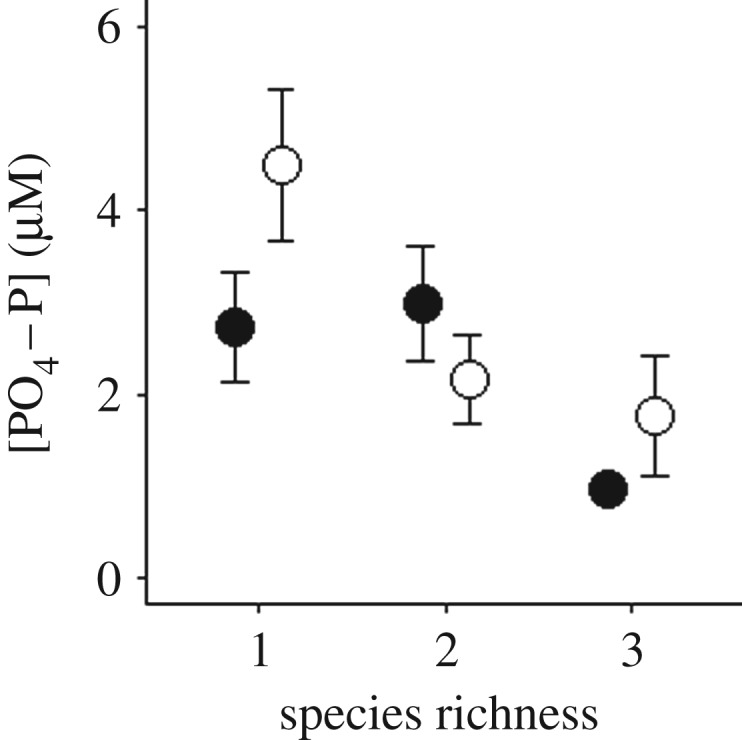
The interactive effects of species richness and compensatory dynamics (present, closed circle; absent, open circle) for uneven (*J*^0.67^) communities on mean (±s.e., *n* = 4) [PO_4_–P].

The analyses confirm that both the sequence in which species are extirpated, the level of species richness and the degree of species evenness are important for both ecosystem process (SBR, ^f-SPI^*L*_mean_, ^f-SPI^*L*_med_, ^f-SPI^*L*_max_, Δ[Br^−^]) and functioning ([NH_4_–N], [NO*_x_*–N], [PO_4_–P]), and that post-extinction community dynamics are particularly influential in determining ecosystem properties. Indeed, when the total biomass of each community was maintained to simulate biomass compensation, the type and extent of particle reworking dramatically altered and subsequently led to changes in nutrient generation. These compensatory effects were stronger in even communities (*J*^1^, figures 2 and 4) relative to those observed for uneven communities (*J*^0.67^, figures 3 and 5).

### (a) Effects on particle reworking and burrow ventilation

In even communities (*J*^1^), SBR was dependent on a three-way interaction between compensatory response × order of extinction × species richness (*L*-ratio = 12.4925, d.f. = 4, *p* = 0.014; electronic supplementary material, model S1; figure 2*a*). Specifically, SBR decreased in non-compensatory communities with decreasing species richness when extinction was ordered by body size or by rarity. In communities with compensatory responses, SBR also decreased with declining species richness when extinction was ordered by body size, but not when extinction was ordered by rarity (figure 2*a*). However, when extinction was random, there was little change in SBR with species richness in both compensatory and non-compensatory communities (figure 2*a*), most likely because species that inhabit or otherwise interact with the sediment–water interface form a distinct functional group and were not preferentially removed from the community. The median depth of particle reworking (^f-SPI^*L*_med_) and the maximum mixed depth of particle reworking (^f-SPI^*L*_max_) were dependent on the interactive effects of compensatory response × order of extinction × species richness (^f-SPI^*L*_med_: *L*-ratio = 32.2030, d.f. = 4, *p* < 0.0001; electronic supplementary material, model S2; figure 2*b*; ^f-SPI^*L*_max_: *L*-ratio = 18.9542, d.f. = 4, *p* = 0.0008; electronic supplementary material, model S3; figure 2*c*). In communities with compensation, ^f-SPI^*L*_med_ decreased when extinction occurred randomly and when ordered by body size, but in communities without compensation, ^f-SPI^*L*_med_ decreased when extinction was ordered by body size or by rarity. Overall, the maximum mixing depth (^f-SPI^*L*_max_) decreased strongly with declining species richness irrespective of extinction scenario, with little difference between communities with and without compensation (figure 2*c*). Burrow ventilation (Δ[Br^−^]) significantly reduced with species richness irrespective of extinction or compensation scenario (*L*-ratio = 6.4222, d.f. = 2, *p* = 0.0403; electronic supplementary material, model S4, figure S1).

For uneven communities that are representative of natural systems (*J*^0.67^), the results revealed that SBR and the median mixed depth of particle reworking was dependent on the interaction compensatory response × order of extinction × species richness (SBR: *L*-ratio = 12.5304, d.f. = 4, *p* = 0.0138; electronic supplementary material, model S8; figure 3*a*; ^f-SPI^*L*_med_: *L*-ratio = 23.8706, d.f. = 4, *p* = 0.0001; electronic supplementary material, model S9; figure 3*b*). Patterns for SBR under random extinction showed a small net decline with decreasing species richness, with a slightly greater decrease in the presence of compensation (figure 3*a*). When extinction was ordered by body size, SBR in both compensatory and non-compensatory communities was highest at intermediate levels of species richness and decreased with species loss (figure 3*a*). By contrast, when extinction was driven by species rarity (figure 3*a*), SBR increased with decreasing species richness for both compensatory and non-compensatory communities. When extinction was random or ordered by rarity, the median mixing depth decreased with species richness in communities without compensation but increased in communities with compensation (figure 3*b*). When extinction was ordered by body size, irrespective of compensatory dynamics, ^f-SPI^*L*_med_ was maintained as species richness declined (figure 3*b*). There was no effect of compensation on the maximum mixed depth of particle reworking or on burrow ventilation, both of which were dependent on an interactive effect of species richness × order of extinction (^f-SPI^*L*_max_: *L*-ratio = 52.8775, d.f. = 4, *p* < 0.0001; electronic supplementary material, model S10; Δ[Br^−^]: *L*-ratio = 16.2130, d.f. = 4, *p* = 0.0027; electronic supplementary material, model S11, figure S2a,b).

### (b) Effects on nutrient generation

In community assemblages with even species distribution, water column nutrient concentrations were affected by the interactive effects of compensatory response × order of extinction ([NH_4_–N]: *L*-ratio = 23.3478, d.f. = 2, *p* < 0.001; electronic supplementary material, model S5; figure 4*a*); [NO*_x_*–N]: *L*-ratio = 7.4958, d.f. = 2, *p* = 0.0236; electronic supplementary material, model S6; figure 4*c*; [PO_4_–P]: *L*-ratio = 8.3114, d.f. = 2, *p* = 0.0157; electronic supplementary material, model S7; figure 4*e*) as well as compensatory response × species richness ([NH_4_–N]: *L*-ratio = 25.4207, d.f. = 2, *p* < 0.001; electronic supplementary material, model S5; figure 4*b*; [NO*_x_*–N]: *L*-ratio = 26.2201, d.f. = 2, *p* < 0.001; electronic supplementary material, model S6; figure 4*d*). In the presence of compensatory dynamics [NH_4_–N] and [NO*_x_*–N] showed similar patterns to one another, irrespective of extinction scenario (figure 4*a*,*c*, respectively); however, in the absence of compensatory dynamics [NH_4_–N] and [NO*_x_*–N] substantively decreased when extinctions were ordered by rarity. For compensatory response × species richness, [NH_4_–N] decreased with species loss when compensation was present (figure 4*b*), while [NO*_x_*–N] increased with decreasing species richness, irrespective of compensation scenario (figure 4*d*).

[PO_4_–P] was highest in communities with compensation when extinction was ordered by body size or rarity, but lowest in the absence of compensatory dynamics when extinction was driven by rarity.

In uneven communities, irrespective of compensation scenario, [NH_4_–N] and [NO*_x_*–N] were dependent on the interactive effects of species richness × order of extinction ([NH_4_–N]: *L*-ratio = 24.6755, d.f. = 4, *p* = 0.0001; electronic supplementary material, model S12, figure S3a; [NO*_x_*–N]: *L*-ratio = 9.78363, d.f. = 2, *p* = 0.0442; electronic supplementary material, model S13, figure S3a). By contrast, [PO_4_–P] was dependent on an interaction between compensatory response × species richness (*L*-ratio = 6.51340, d.f. = 2, *p* = 0.0385; electronic supplementary material, model S14; figure 5). Overall, [PO_4_–P] increased with decreasing species richness and was higher in the presence of compensatory dynamics (figure 5).

## 4. Discussion

Our study provides empirical evidence, consistent with the predictions of recent trait-based simulations of species loss [25], that the ecosystem consequences of extinction can be fundamentally altered by compensatory responses within the surviving community. However, we find that the strength of such a response is contingent on compositional differences that arise from the order of species loss and the number of species remaining in the post-extinction community [26,27,58]. Further, we find that the effects of biomass compensation, although not as strong as anticipated, are less prominent in uneven communities that have a structure typical of natural communities than in communities with an even species distribution, as per the archetypal design of biodiversity–ecosystem function experiments [22]. This distinction is important because a majority of experimental manipulations of biodiversity fall short of allowing community dynamics and compensatory responses to fully develop [59,60], reducing the likelihood of conveying the most likely community response to extinction for a natural setting [61,62]. Recent work has shown that adjustments to community structure in the absence of species loss can have consequential effects on ecosystem functioning that relate to the rank order of species dominance [49], rather than dominant species identity [63,64], and changes in species density and biomass [65–69]. Such transient changes in how dominance and identity are represented as communities respond to forcing over time [70] have important ramifications for the design and analysis of contemporary biodiversity experiments [71,72], as well as the relevance of their findings for practical application [73]. The results also reinforce the role of species trait identity and variability [74] as major determinants of ecosystem functioning [22].

At a broader ecological level, our findings indicate that the ecological consequences of extinction are unlikely to meet expectation (i.e. some form of a positive but decelerating curve [22,23]) when projections are based on pre-extinction community properties and dynamics [75]. This is because the type, timing and severity of extinction generates a legacy that influences the capacity of, and way in which, the surviving community will respond and affect ecosystem properties. The complexities of how species respond to novel circumstances are difficult to anticipate and are yet to be fully explored, even in relation to near-term aspects of climate change [76]. However, understanding variability in species responses to abiotic and biotic change [77], as well as the context-dependent contributions they make to ecosystem functioning over time [59,78,79], will help to refine the likelihood of various ecological outcomes against specific scenarios. Here, biomass compensation had a positive effect on sediment reworking in even communities, especially at intermediate levels of species richness, while incidences of over-compensation were particularly pronounced at high levels of species richness [80]. For uneven communities, functioning was only maintained when extinctions were random or ordered by rarity. However, when extinction was driven by body size in both even and uneven communities, pre-extinction levels of sediment mixing could not be maintained in the surviving community, regardless of the identity and ordering of the compensating species because smaller species contribute little to bioturbation [26,41]. By contrast, where species shared physiological and/or behavioural traits across the species pool, as was the case for SBR, functioning was generally maintained across the species richness gradient. Hence, the presence and expression of species traits dictate how the surviving community moderate ecosystem properties, and explain why ordered species extinctions can result in no change in some functions and large changes in other functions [26,49], but these patterns can be further modified by compensatory adjustments to the assemblage.

Our findings are in broad agreement with previous hypotheses which state that the potential or probability for compensatory dynamics countering the consequences of biodiversity loss will depend on the level of functional redundancy within a system [80,81], but they acknowledge the importance of species that exhibit low or different effect trait values in maintaining ecosystems as circumstances change. Ultimately, the net ecosystem response is a multiple of the role of which species survive and the population response to perturbation, including the sequence of species loss [82]. We find that the effect of extinction order is driven by species-specific differences within a community, and especially the disproportionate effect of *H. diversicolor* on the depth of particle mixing and the inability of the mud snail *P. ulvae* to replace the loss of bioturbation activity previously performed by other species [38,51,83]. While this demonstrates that compensatory effects are not always able to buffer the changes in ecosystem processes and functions associated with species loss [84], our data suggest that, on average, compensatory mechanisms will be sufficient to reduce, in whole or in part, the ecological consequences of species loss. Indeed, nutrient release was either maintained or increased in the presence of compensation, even when extinction was driven by body size, and there is some evidence to suggest that other mechanisms may lead to over-compensation prior to the development of community dynamics over the longer term. Higher levels of functional redundancy, for example, will be particularly important as circumstances change and may lessen the likelihood and/or magnitude of unstable fluctuations in ecosystem properties. Although the present study was unable to account for processes that act over longer time scales, such as adaptation [85,86] and evolutionary change [87,88], our findings suggest that an immediate challenge is to determine the circumstances under which species exhibit compensatory responses (e.g. [21]) and whether or not the presence of compensatory processes refine understanding of biodiversity–function relations. In the meantime, we advocate that management efforts should prioritize the conservation of species based on their contribution to maintaining multiple ecosystem processes and functions. In doing so, it will be important to recognize that the compensatory capacity of a community is dynamic and will respond to changes in biological and environmental context [89] that, in turn, are likely to lead to a wider range of ecological outcomes than are presently appreciated.

## Supplementary Material

Supporting data, statistical summaries and experimental data

## Data accessibility

All data are provided in the electronic supplementary material, Data S1.
