# Supplementary material for: Compensatory responses can alter the form of the biodiversity–function relation curve
Source: Proc Biol Sci. 2019 Apr 17;286(1901):20190287. doi: 10.1098/rspb.2019.0287 (PMC6501933; doi:10.1098/rspb.2019.0287)
Supplement: Supporting data, statistical summaries and experimental data [file rspb20190287supp1.docx]

**Electronic supplementary material**

**Compensatory responses can alter the form of the biodiversity-function relation curve**

Matthias S. Thomsen^1*^, Jasmin A. Godbold^1,2^, Clement Garcia^3^, Stefan G. Bolam^3^, Ruth Parker^3^ & Martin Solan^1^

^1^Ocean and Earth Science, National Oceanography Centre Southampton, University of Southampton, Waterfront Campus, European Way, Southampton. SO14 3ZH, UK.

^2^Biological Sciences, University of Southampton, Highfield Campus, Southampton. SO17 1BJ, UK.

^3^Centre for Environment, Fisheries and Aquaculture Science (Cefas), Pakefield Road, Lowestoft, Suffolk, NR33 0HT, UK.

ORCID: MST, 0000-0002-9017-3997; CG, 0000-0001-5916-8914; SGB; 0000-0001-6604-4741, RP, 0000-0003-4130-5997; JAG, 0000-0001-5558-8188; MS, 0000-0001-9924-5574

*Author for correspondence: Matthias S. Thomsen, e-mail: mst1f13@soton.ac.uk


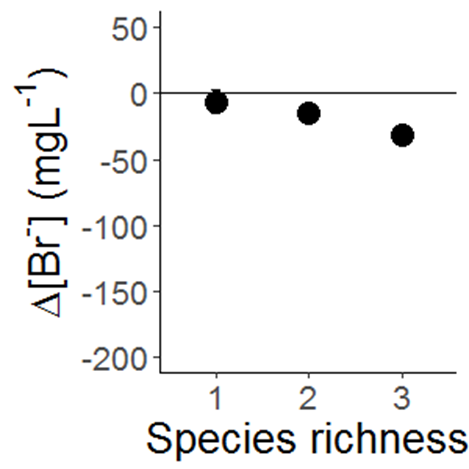


**Figure S1.** Burrow ventilation ([Br] Δ, mg L^-1^, mean ± s.e., n = 4) in even (J^1.00^) community assemblages across levels of species richness.





**Figure S2.** Effects of species richness and extinction scenario (random [white], body size [black], rarity [grey]) in natural (J^0.67^) community assemblages on maximum mixing depth of particle reworking (^f-SPI^L_max_, cm, mean ± s.e., n = 4) and burrow ventilation (Δ[Br^-^], mg L^-1^, mean ± s.e., n = 4).





**Figure S3.** Effects of species richness and extinction scenario (random [white], body size [black], rarity [grey]) in natural (J^0.67^) community assemblages on NH_4_-N and NO_x_-N concentrations ([NH_4_-N],[NO_x_-N], μM, mean ± s.e., n = 4).

**Table S1.** The distribution of biomass between species for each scenario of extinction (random, body size, rarity) and compensatory response (NoComp, no compensation; Comp, full compensation) in even communities (J^1^). All permutations were replicated (n = 4). Controls containing no macrofauna are not included.

| Extinction scenario and response | Community assembly | Total biomass | *Peringia ulvae (P)* | *Corophium volutator (C)* | *Hediste*  *diversicolor* *(H)* | |
| --- | --- | --- | --- | --- | --- | --- |
| Random × NoComp | P | 0.667 | 0.667 | 0.000 | 0.000 |  |
| Random × NoComp | C | 0.667 | 0.000 | 0.667 | 0.000 |  |
| Random × NoComp | H | 0.667 | 0.000 | 0.000 | 0.667 |  |
| Random × NoComp | PC | 1.333 | 0.667 | 0.667 | 0.000 |  |
| Random × NoComp | PH | 1.333 | 0.667 | 0.000 | 0.667 |  |
| Random × NoComp | HC | 1.333 | 0.000 | 0.667 | 0.667 |  |
| Random × NoComp | PCH | 2.000 | 0.667 | 0.667 | 0.667 |  |
| Random × Comp | H | 2.000 | 2.000 | 0.000 | 0.000 |  |
| Random × Comp | C | 2.000 | 0.000 | 2.000 | 0.000 |  |
| Random × Comp | H | 2.000 | 0.000 | 0.000 | 2.000 |  |
| Random × Comp | PC | 2.000 | 1.000 | 1.000 | 0.000 |  |
| Random × Comp | PH | 2.000 | 1.000 | 0.000 | 1.000 |  |
| Random × Comp | HC | 2.000 | 0.000 | 1.000 | 1.000 |  |
| Random × Comp | PCH | 2.000 | 0.667 | 0.667 | 0.667 |  |
| Body size × NoComp | PCH | 2.000 | 0.667 | 0.667 | 0.667 |  |
| Body size × NoComp | PC | 1.333 | 0.667 | 0.667 | 0.000 |  |
| Body size × NoComp | P | 0.667 | 0.667 | 0.000 | 0.000 |  |
| Body size × Comp | PCH | 2.000 | 0.667 | 0.667 | 0.667 |  |
| Body size × Comp | PC | 2.000 | 1.000 | 1.000 | 0.000 |  |
| Body size × Comp | P | 2.000 | 2.000 | 0.000 | 0.000 |  |
| Rarity × NoComp | PCH | 2.000 | 0.667 | 0.667 | 0.667 |  |
| Rarity × NoComp | PC | 1.333 | 0.667 | 0.667 | 0.000 |  |
| Rarity × NoComp | C | 0.667 | 0.000 | 0.667 | 0.000 |  |
| Rarity × Comp | PCH | 2.000 | 0.667 | 0.667 | 0.667 |  |
| Rarity × Comp | PC | 2.000 | 1.000 | 1.000 | 0.000 |  |
| Rarity × Comp | C | 2.000 | 0.000 | 2.000 | 0.000 |  |

**Table S2.** Experimental design for each scenario of extinction and compensatory response (n=4) and community assembly and biomass in communities representative of natural evenness (J^0.67^). Controls (n=4) not included in this table.

| Extinction scenario and response | Community assembly | Total biomass | *Peringia ulvae (P)* | *Corophium volutator (C)* | *Hediste*  *diversicolor (H)* |
| --- | --- | --- | --- | --- | --- |
| Random × NoComp | P | 1.584 | 1.584 | 0.000 | 0.000 |
| Random × NoComp | C | 0.101 | 0.000 | 0.101 | 0.000 |
| Random × NoComp | H | 0.144 | 0.000 | 0.000 | 0.144 |
| Random × NoComp | PC | 1.685 | 1.584 | 0.101 | 0.000 |
| Random × NoComp | PH | 1.728 | 1.584 | 0.000 | 0.144 |
| Random × NoComp | HC | 0.245 | 0.000 | 0.101 | 0.144 |
| Random × NoComp | PCH | 1.829 | 1.584 | 0.101 | 0.144 |
| Random × Comp | P | 1.829 | 1.829 | 0.000 | 0.000 |
| Random × Comp | C | 1.829 | 0.000 | 1.829 | 0.000 |
| Random × Comp | H | 1.829 | 0.000 | 0.000 | 1.829 |
| Random × Comp | PC | 1.829 | 1.656 | 0.173 | 0.000 |
| Random × Comp | PH | 1.829 | 1.635 | 0.000 | 1.194 |
| Random × Comp | HC | 1.829 | 0.000 | 0.893 | 0.936 |
| Random × Comp | PCH | 1.829 | 1.584 | 0.101 | 0.144 |
| Body size × NoComp | PCH | 1.829 | 1.584 | 0.101 | 0.144 |
| Body size × NoComp | PC | 1.685 | 1.584 | 0.101 | 0.000 |
| Body size × NoComp | P | 1.584 | 1.584 | 0.000 | 0.000 |
| Body size × Comp | PCH | 1.829 | 1.584 | 0.101 | 0.144 |
| Body size × Comp | PC | 1.829 | 1.656 | 0.173 | 0.000 |
| Body size × Comp | P | 1.829 | 1.829 | 0.000 | 0.000 |
| Rarity × NoComp | PCH | 1.829 | 1.584 | 0.101 | 0.144 |
| Rarity × NoComp | PC | 1.685 | 1.584 | 0.101 | 0.000 |
| Rarity × NoComp | C | 0.101 | 0.000 | 0.101 | 0.000 |
| Rarity × Comp | PCH | 1.829 | 1.584 | 0.101 | 0.144 |
| Rarity × Comp | PC | 1.829 | 1.656 | 0.173 | 0.000 |
| Rarity × Comp | C | 1.829 | 0.000 | 1.829 | 0.000 |

**Statistical model summary**

Summary of statistical models (Model S1 to S14), for even communities (Model S1 to S7) and uneven communities (Model S8 to S14). For each model, we list the initial linear regression model and the minimal adequate model. As the experiment could not be assembled simultaneously, the timing of each run forms a random factor (run). Where it was necessary to account for violation of homogeneity of variance, we used a linear regression with GLS estimation. Hence, where appropriate, we provide a summary of the coefficient table. The coefficients indicate the relative performance of each level relative to the baseline, as indicated. Coefficients ± SE, t-values and significance values are presented.

**Statistical models for the effect of compensation and extinction order**

**(i) Even (J^1^) communities**

**Model S1 |** Surface boundary roughness (SBR, cm)

Initial linear regression model:

lme(SBR~Compensation+Extinction+

Compensation:Extinction,

random = ~1|as.factor(Run),

method = "ML")

No minimal adequate model, intercept only (L-ratio = 4.1847, d.f. = 2, p = 0.1234).

**Model S2 |** Median maximum mixed depth of particle reworking (^f-SPI^L_med_, cm)

Initial linear regression model:

lme(^f-SPI^L_med_ ~ Compensation+Extinction+

Compensation:Extinction,

random = ~1|as.factor(Run),

weights = varIdent(form=~1|Extinction*Compensation),

method = "ML")

Minimal adequate model:

lme(^f-SPI^L_med_ ~ Compensation+Extinction,

random = ~1|as.factor(Run),

weights = varIdent(form=~1|Extinction*Compensation),

method = "ML")

|  | d.f. | AIC | L-Ratio | p-value |
| --- | --- | --- | --- | --- |
| Full model | 11 | -6.313776 |  |  |
| Compensation | 8 | 0.081480 | 8.395256 | 0.0038 |
| Extinction | 9 | 3.638944 | 13.95272 | 9e-04 |

Coefficient Table

Intercept ± SE (when baseline is for random): 0.7062 ± 0.0439, t = 16.083, p = 0.0000.

| Bodysize | Bodysize |  |  |
| --- | --- | --- | --- |
| Random | 0.0039 ± 0.0497  0.0780  (0.9380) | Random |  |
| Rarity | 0.1526 ± 0.0492  3.103  **(0.0025)** | 0.1564 ± 0.0432  3.6231  **(0.0005)** | Rarity |

**Model S3 |** Maximum mixed depth of particle reworking (^f-SPI^L_max_, cm)

Initial linear regression model:

lme(^f-SPI^L_max_ ~ Compensation+Extinction+

Compensation:Extinction,

random = ~1|as.factor(Run),

method = "ML")

Minimal adequate model:

lme(^f-SPI^L_max_ ~Extinction,

random = ~1|as.factor(Run),

method = "ML")

|  | d.f | AIC | L-Ratio | p-value |
| --- | --- | --- | --- | --- |
| Full model | 5 | 602.7047 |  |  |
| Extinction | 3 | 605.1285 | 6.423848 | 0.0403 |

Coefficient Table

Intercept ± SE (when baseline is for Random): 7.2715 ± 0.5670, t = 12.825, p = 0.0000.

| Bodysize | Bodysize |  |  |
| --- | --- | --- | --- |
| Random | 2.2838 ± 1.0352  2.206  **(0.0297)** | Random |  |
| Rarity | 0.3623 ± 1.2248  0.2958  (0.7680) | 1.9215± 1.0352  1.8563  (0.0664) | Rarity |

**Model S4 |** Ammonium concentration (NH_4_-N, µM)

Initial linear regression model:

lme(NH_4_-N ~Compensation+Extinction+

Compensation:Extinction,

random = ~1|as.factor(Run),

weights = varIdent(form=~1|Extinction*Compensation),

method = "ML")

Minimal adequate model:

lme(NH_4_-N~Compensation+Extinction+

Compensation:Extinction,

random = ~1|as.factor(Run),

weights = varIdent(form=~1|Extinction*Compensation),

method = "ML")

|  | d.f | AIC | L-Ratio | p-value |
| --- | --- | --- | --- | --- |
| Full model | 13 | 1151.580 |  |  |
| Compensation | 5 | 1171.858 | 26.27831 | <.0001 |
| Extinction | 4 | 1173.514 | 29.93443 | <.0001 |
| Compensation:Extinction | 11 | 1173.856 | 26.27567 | <.0001 |

Coefficient Table

Intercept ± SE (when baseline is for Random): 254.5726 ± 20.5701, t = 12.3759, p = 0.0000.

| Bodysize | Bodysize |  |  |
| --- | --- | --- | --- |
| Random | 73.9026 ± 26.8884  -2.7485  **(0.0072)** | Random |  |
| Rarity | 177.9345 ± 17.3182  10.2744  **(0.0000)** | 251.8370 ± 20.5716  12.2420  **(0.0000)** | Rarity |

**Model S5 |** Nitrate + nitrite concentration (NO_x_-N, µM)

Initial linear regression model:

lme(NO_x_-N~Compensation+Extinction+

Compensation:Extinction,

random = ~1|as.factor(Run),

weights = varIdent(form=~1|Extinction*Compensation),

method = "ML")

Minimal adequate model:

lme(NO_x_-N ~Compensation+Extinction+

Compensation:Extinction,

random = ~1|as.factor(Run),

weights = varIdent(form=~1|Extinction*Compensation),

method = "ML")

|  | d.f | AIC | L-Ratio | p-value |
| --- | --- | --- | --- | --- |
| Full model | 13 | 891.5928 |  |  |
| Compensation | 10 | 911.4338 | 25.84106 | <.0001 |
| Extinction | 9 | 910.4265 | 26.83378 | <.0001 |
| Compensation:Extinction | 11 | 912.7062 | 25.11348 | <.0001 |

**Model S6 |** Phosphate concentration (PO_4_-P, µM)

Initial linear regression model:

lme(PO_4_-P ~ Compensation+Extinction+

Compensation:Extinction,

random = ~1|as.factor(Run),

method = "ML")

Minimal adequate model:

lme(PO_4_-P ~ Compensation+Extinction+

Compensation:Extinction,

random = ~1|as.factor(Run),

method = "ML")

|  | d.f | AIC | L-Ratio | p-value |
| --- | --- | --- | --- | --- |
| Full model | 13 | 426.4792 |  |  |
| Compensation | 10 | 441.0710 | 20.59183 | 1e-04 |
| Extinction | 9 | 439.6768 | 21.19764 | 3e-04 |
| Compensation:Extinction | 11 | 430.7855 | 8.306257 | 0.0157 |

**Model S7 |** Bromide concentration (Br, mg l^-1^)

Initial linear regression model:

lme(Bromide~Compensation+Extinction+

Compensation:Extinction,

random = ~1|as.factor(Run),

method = "ML")

No minimal adequate model, intercept only only (L-ratio = 1.376575, d.f. = 2, p = 0.5024).

**(ii) Uneven (J^0.67^) communities**

**Model S8 |** Surface boundary roughness (SBR, cm)

Initial linear regression model:

lme(SBR~Compensation+Extinction+

Compensation:Extinction,

random = ~1|as.factor(Run),

weights = varIdent(form=~1|Extinction),

method = "ML")

No minimal adequate model, intercept only (L-ratio = 0.8632205, d.f. = 1, p = 0.3528).

**Model S9 |** Median maximum mixed depth of particle reworking (^f-SPI^L_med_, cm)

Initial linear regression model:

lme(^f-SPI^L_med_ ~ Compensation+Extinction+

Compensation:Extinction,

random = ~1|as.factor(Run),

weights = varIdent(form=~1|Extinction*Compensation),

method = "ML")

Minimal adequate model:

lme(^f-SPI^L_med_ ~ Compensation+Extinction+

Compensation:Extinction,

random = ~1|as.factor(Run),

weights = varIdent(form=~1|Extinction*Compensation),

method = "ML")

|  | d.f | AIC | L-Ratio | p-value |
| --- | --- | --- | --- | --- |
| Full model | 13 | -113.01783 |  |  |
| Compensation | 5 | -100.1075 | 18.91028 | 3e-04 |
| Extinction | 9 | -99.22558 | 21.79225 | 2e-04 |
| Compensation:Extinction | 11 | -98.33025 | 18.68758 | 1e-04 |

**Model S10 |** Maximum mixed depth of particle reworking (^f-SPI^L_max_, cm)

Initial linear regression model:

lme(^f-SPI^L_max_ ~ Compensation+Extinction+

Compensation:Extinction,

random = ~1|as.factor(Run),

method = "ML")

Minimal adequate model:

lme(^f-SPI^L_max_ ~Extinction,

random = ~1|as.factor(Run),

method = "ML")

|  | d.f | AIC | L-Ratio | p-value |
| --- | --- | --- | --- | --- |
| Full model | 5 | 602.3072 |  |  |
| Extinction | 3 | 605.1449 | 6.837739 | 0.0327 |

Coefficient Table

Intercept ± SE (when baseline is for Random): 6.2223 ± 1.2318, t = 5.0515, p = 0.0000.

| Bodysize | Bodysize |  |  |
| --- | --- | --- | --- |
| Random | 2.2279 ± 1.0150  2.195  **(0.0305)** | Random |  |
| Rarity | 0.8104 ± 1.2855  0.6305  (0.5298) | 1.4174 ± 1.1140  1.2723  (0.2062) | Rarity |

**Model S11 |** Ammonium concentration (NH_4_-N, µM)

Initial linear regression model:

lme(NH_4_-N ~Compensation+Extinction+

Compensation:Extinction,

random = ~1|as.factor(Run),

weights = varIdent(form=~1|Extinction*Compensation),

method = "ML")

Minimal adequate model:

lme(NH_4_-N ~Extinction,

random = ~1|as.factor(Run),

weights = varIdent(form=~1|Extinction*Compensation),

method = "ML")

|  | d.f | AIC | L-Ratio | p-value |
| --- | --- | --- | --- | --- |
| Full model | 10 | 1211.747 |  |  |
| Extinction | 8 | 1213.807 | 6.059796 | 0.0483 |

Coefficient Table

Intercept ± SE (when baseline is for Random): 221.50584 ± 27.19878, t = 8.143963, p = 0.0000.

| Bodysize | Bodysize |  |  |
| --- | --- | --- | --- |
| Random | 31.3621 ± 13.2623  2.3648  **(0.0200)** | Random |  |
| Rarity | 9.9464 ± 21.7136  0.4581  (0.6479) | 21.4157 ± 22.0989  0.9691  (0.3349) | Rarity |

**Model S12 |** Nitrate + nitrite concentration (NO_x_-N, µM)

Initial linear regression model:

lme(NO_x_-N~ Compensation+Extinction+

Compensation:Extinction,

random = ~1|as.factor(Run),

weights = varIdent(form=~1|Extinction*Compensation),

method = "ML")

Minimal adequate model:

lme(NO_x_-N ~Extinction,

random = ~1|as.factor(Run),

weights = varIdent(form=~1|Extinction*Compensation),

method = "ML")

|  | d.f | AIC | L-Ratio | p-value |
| --- | --- | --- | --- | --- |
| Full model | 10 | 939.2344 |  |  |
| Extinction | 8 | 943.3458 | 8.111428 | 0.0173 |

Coefficient Table

Intercept ± SE (when baseline is for Random): 51.57836 ± 4.167782, t = 12.375494, p = 0.0000.

| Bodysize | Bodysize |  |  |
| --- | --- | --- | --- |
| Random | 6.5217 ± 3.0130  2.1645  **(0.0328)** | Random |  |
| Rarity | 7.8774 ± 5.6148  1.4029  (0.1638) | 14.3991 ± 5.6359  2.5549  **(0.0121)** | Rarity |

**Model S13 |** Phosphate concentration (PO_4_-P, µM)

Initial linear regression model:

lme(PO4~Compensation+Extinction+

Compensation:Extinction,

random = ~1|as.factor(Run),

weights = varIdent(form=~1|Extinction*Compensation),

method = "ML")

Minimal adequate model:

lme(PO_4_-P ~Extinction,

random = ~1|as.factor(Run),

weights = varIdent(form=~1|Extinction*Compensation),

method = "ML")

|  | d.f | AIC | L-Ratio | p-value |
| --- | --- | --- | --- | --- |
| Full model | 10 | 497.0147 |  |  |
| Extinction | 8 | 505.8557 | 12.841 | 0.0016 |

Coefficient Table

Intercept ± SE (when baseline is for Random): 3.218481 ± 5.159960, t = 12.3759, p = 0.0000.

| Bodysize | Bodysize |  |  |
| --- | --- | --- | --- |
| Random | 0.3212 ± 0.7557  0.4251  (0.6717) | Random |  |
| Rarity | 1.8137 ± 0.6568  2.7617  **(0.0069)** | 1.4925 ± 0.4370  3.4154  **(0.0009)** | Rarity |

**Model S14 |** Bromide concentration (Br, µM)

Initial linear regression model:

lme(Bromide~Compensation,

random = ~1|as.factor(Run),

weights = varIdent(form=~1|Extinction*Compensation),

method = "ML")

No minimal adequate model, intercept only (L-ratio = 3.83717, d.f. = 2, p = 0.1468).

**Data S1 |** Summary of data used for statistical analysis. Data in the absence of macrofauna is shown for comparison but was not included in the statistical analyses. Treatment = compensatory response (Comp = full biomass compensation, NoComp = no biomass compensation), Species = Community composition (H = *Hediste diversicolor*, P = *Peringia ulvae*, C = *Corophium volutator*), Run = experimental run.

| Extinction | Treatment | Species | Evenness (*J*) | Total biomass | Run | Repli-cate | SBR (cm) | ^f-SPI^ L_med_  (cm) | ^f-SPI^ L_max_ (cm) | Δ[Br^-^] (mg L^-1^) | [NO_X_-N]  (µM) | [NH_4_-N]  (µM) | [PO_4_-P] (µM) |
| --- | --- | --- | --- | --- | --- | --- | --- | --- | --- | --- | --- | --- | --- |
| Bodysize | Comp | P | 0.67 | 1.829 | 2 | 1 | 0.396 | 0.433 | 1.067 | 15.582 | 56.650 | 180.171 | 1.107 |
| Bodysize | Comp | P | 0.67 | 1.829 | 2 | 2 | 0.314 | 0.433 | 1.004 | -16.298 | 58.441 | 197.454 | 1.756 |
| Bodysize | Comp | P | 0.67 | 1.829 | 2 | 3 | 0.433 | 0.452 | 0.785 | 47.651 | 52.048 | 210.474 | 8.940 |
| Bodysize | Comp | P | 0.67 | 1.829 | 2 | 4 | 0.274 | 0.427 | 1.023 | -8.612 | 77.150 | 122.899 | 3.631 |
| Bodysize | Comp | PC | 0.67 | 1.829 | 2 | 1 | 0.511 | 0.414 | 3.409 | 0.523 | 85.856 | 104.664 | 1.827 |
| Bodysize | Comp | PC | 0.67 | 1.829 | 1 | 2 | 0.697 | 0.483 | 2.022 | 8.773 | 61.889 | 200.098 | 8.758 |
| Bodysize | Comp | PC | 0.67 | 1.829 | 1 | 3 | 0.709 | 0.477 | 2.285 | -31.754 | 46.488 | 212.224 | 1.597 |
| Bodysize | Comp | PC | 0.67 | 1.829 | 1 | 4 | 0.748 | 0.414 | 3.296 | -11.141 | 55.585 | 130.412 | 1.791 |
| Bodysize | Comp | PCH | 0.67 | 1.829 | 1 | 1 | 0.480 | 0.534 | 11.162 | -21.901 | 45.340 | 165.979 | 1.357 |
| Bodysize | Comp | PCH | 0.67 | 1.829 | 2 | 2 | 0.523 | 0.458 | 10.874 | -26.254 | 57.606 | 173.117 | 1.175 |
| Bodysize | Comp | PCH | 0.67 | 1.829 | 1 | 3 | 0.617 | 0.439 | 10.754 | 7.052 | 45.089 | 189.760 | 0.383 |
| Bodysize | Comp | PCH | 0.67 | 1.829 | 2 | 4 | 0.810 | 0.458 | 10.108 | -5.509 | 60.565 | 154.674 | 1.901 |
| Bodysize | NoComp | P | 0.67 | 1.584 | 1 | 1 | 0.386 | 0.433 | 0.942 | -12.148 | 79.724 | 100.185 | 3.095 |
| Bodysize | NoComp | P | 0.67 | 1.584 | 2 | 2 | 0.402 | 0.452 | 0.766 | -54.998 | 60.655 | 119.833 | 0.007 |
| Bodysize | NoComp | P | 0.67 | 1.584 | 2 | 3 | 0.408 | 0.452 | 0.954 | 67.582 | 57.353 | 197.615 | 9.202 |
| Bodysize | NoComp | P | 0.67 | 1.584 | 1 | 4 | 0.458 | 0.433 | 1.017 | 23.691 | 57.335 | 229.451 | 9.471 |
| Bodysize | NoComp | PC | 0.67 | 1.685 | 1 | 1 | 0.986 | 0.458 | 1.764 | -55.578 | 52.451 | 193.073 | 1.671 |
| Bodysize | NoComp | PC | 0.67 | 1.685 | 2 | 2 | 0.723 | 0.515 | 2.241 | 5.785 | 78.630 | 81.598 | 1.318 |
| Bodysize | NoComp | PC | 0.67 | 1.685 | 1 | 3 | 0.960 | 0.596 | 2.888 | -1.628 | 60.830 | 101.526 | 1.642 |
| Bodysize | NoComp | PC | 0.67 | 1.685 | 2 | 4 | 0.860 | 0.433 | 4.307 | -16.222 | 53.695 | 148.821 | 2.019 |
| Bodysize | NoComp | PCH | 0.67 | 1.829 | 1 | 1 | 0.654 | 0.502 | 11.181 | -15.919 | 56.476 | 164.560 | 1.134 |
| Bodysize | NoComp | PCH | 0.67 | 1.829 | 1 | 2 | 0.660 | 0.603 | 10.767 | -18.405 | 54.030 | 135.372 | 1.320 |
| Bodysize | NoComp | PCH | 0.67 | 1.829 | 1 | 3 | 0.436 | 0.414 | 10.918 | -26.690 | 50.552 | 197.546 | 1.515 |
| Bodysize | NoComp | PCH | 0.67 | 1.829 | 1 | 4 | 0.377 | 0.402 | 11.100 | -2.454 | 45.230 | 330.927 | 8.879 |
| Random | Comp | C | 0.67 | 1.829 | 1 | 1 | 0.592 | 1.249 | 3.930 | -43.426 | 165.871 | 72.549 | 2.155 |
| Random | Comp | C | 0.67 | 1.829 | 2 | 2 | 0.885 | 0.954 | 3.622 | 58.391 | 119.299 | 129.909 | 2.233 |
| Random | Comp | C | 0.67 | 1.829 | 1 | 3 | 0.461 | 1.425 | 3.629 | -8.263 | 177.907 | 19.847 | 2.144 |
| Random | Comp | C | 0.67 | 1.829 | 1 | 4 | 1.103 | 0.458 | 4.495 | -18.647 | 144.191 | 58.682 | 2.165 |
| Random | Comp | P | 0.67 | 1.829 | 1 | 1 | 0.339 | 0.439 | 0.879 | -41.813 | 56.290 | 145.860 | 0.343 |
| Random | Comp | P | 0.67 | 1.829 | 1 | 2 | 0.548 | 0.465 | 0.973 | -16.612 | 77.277 | 116.929 | 3.092 |
| Random | Comp | P | 0.67 | 1.829 | 1 | 3 | 0.287 | 0.427 | 0.979 | -30.730 | 73.349 | 157.188 | 3.625 |
| Random | Comp | P | 0.67 | 1.829 | 2 | 4 | 0.301 | 0.414 | 1.092 | 24.556 | 58.337 | 206.452 | 9.351 |
| Random | Comp | PC | 0.67 | 1.829 | 1 | 1 | 0.716 | 0.396 | 1.532 | 14.093 | 68.157 | 220.312 | 1.646 |
| Random | Comp | PC | 0.67 | 1.829 | 2 | 2 | 0.660 | 0.477 | 3.095 | 4.300 | 82.867 | 91.662 | 1.307 |
| Random | Comp | PC | 0.67 | 1.829 | 1 | 3 | 0.634 | 0.490 | 2.725 | -6.477 | 48.360 | 233.088 | 8.844 |
| Random | Comp | PC | 0.67 | 1.829 | 1 | 4 | 0.665 | 0.458 | 3.924 | -24.631 | 55.862 | 211.776 | 1.829 |
| Random | Comp | PCH | 0.67 | 1.829 | 2 | 1 | 1.078 | 0.521 | 11.150 | 6.213 | 66.435 | 172.891 | 1.404 |
| Random | Comp | PCH | 0.67 | 1.829 | 2 | 2 | 0.698 | 0.465 | 11.709 | -28.384 | 36.007 | 253.330 | 0.773 |
| Random | Comp | PCH | 0.67 | 1.829 | 1 | 3 | 0.559 | 0.433 | 10.893 | 3.970 | 31.956 | 300.685 | 0.670 |
| Random | Comp | PCH | 0.67 | 1.829 | 2 | 4 | 0.586 | 0.515 | 11.006 | -4.039 | 44.004 | 197.652 | 0.741 |
| Random | Comp | PH | 0.67 | 1.829 | 2 | 1 | 0.471 | 0.433 | 10.610 | -38.327 | 37.381 | 266.140 | 0.603 |
| Random | Comp | PH | 0.67 | 1.829 | 1 | 2 | 0.521 | 0.421 | 10.742 | -5.851 | 28.226 | 327.998 | 7.777 |
| Random | Comp | PH | 0.67 | 1.829 | 2 | 3 | 0.399 | 0.471 | 10.397 | 14.785 | 93.482 | 299.074 | 1.301 |
| Random | Comp | PH | 0.67 | 1.829 | 2 | 4 | 0.540 | 0.458 | 9.932 | -48.823 | 47.700 | 343.413 | 0.978 |
| Random | Comp | H | 0.67 | 1.829 | 1 | 1 | 0.753 | 0.389 | 10.698 | -35.693 | 6.128 | 469.898 | 7.226 |
| Random | Comp | H | 0.67 | 1.829 | 2 | 2 | 0.622 | 0.364 | 10.541 | -21.930 | 7.277 | 465.058 | 0.173 |
| Random | Comp | H | 0.67 | 1.829 | 1 | 3 | 0.542 | 0.502 | 10.836 | -62.155 | 8.461 | 260.168 | 0.107 |
| Random | Comp | H | 0.67 | 1.829 | 1 | 4 | 0.710 | 0.339 | 10.710 | -65.707 | 10.423 | 265.261 | 0.272 |
| Random | Comp | HC | 0.67 | 1.829 | 1 | 1 | 0.710 | 0.540 | 11.351 | -37.011 | 42.655 | 301.520 | 1.153 |
| Random | Comp | HC | 0.67 | 1.829 | 1 | 2 | 1.469 | 0.634 | 11.181 | -17.881 | 57.056 | 399.439 | 7.685 |
| Random | Comp | HC | 0.67 | 1.829 | 2 | 3 | 0.617 | 0.778 | 9.819 | -42.219 | 66.122 | 225.665 | 1.044 |
| Random | Comp | HC | 0.67 | 1.829 | 2 | 4 | 1.047 | 0.678 | 10.403 | -28.163 | 56.828 | 248.752 | 0.897 |
| Random | NoComp | C | 0.67 | 0.101 | 1 | 1 | 0.684 | 0.248 | 2.963 | 8.748 | 60.133 | 155.156 | 8.271 |
| Random | NoComp | C | 0.67 | 0.101 | 1 | 2 | 0.748 | 0.289 | 4.326 | 19.837 | 79.891 | 101.537 | 1.430 |
| Random | NoComp | C | 0.67 | 0.101 | 2 | 3 | 0.402 | 0.094 | 1.839 | -37.329 | 62.440 | 179.507 | 1.203 |
| Random | NoComp | C | 0.67 | 0.101 | 2 | 4 | 0.530 | 0.283 | 0.810 | -53.806 | 72.127 | 98.453 | 1.598 |
| Random | NoComp | P | 0.67 | 1.584 | 1 | 1 | 0.345 | 0.427 | 0.910 | 17.617 | 56.372 | 204.938 | 8.328 |
| Random | NoComp | P | 0.67 | 1.584 | 2 | 2 | 0.439 | 0.396 | 0.766 | 55.127 | 55.903 | 220.440 | 9.016 |
| Random | NoComp | P | 0.67 | 1.584 | 2 | 3 | 0.283 | 0.446 | 0.910 | -27.582 | 58.237 | 216.766 | 9.334 |
| Random | NoComp | P | 0.67 | 1.584 | 1 | 4 | 0.207 | 0.446 | 1.017 | 19.062 | 57.303 | 192.141 | 1.294 |
| Random | NoComp | PC | 0.67 | 1.685 | 2 | 1 | 0.692 | 0.534 | 2.737 | 16.629 | 70.071 | 100.475 | 1.334 |
| Random | NoComp | PC | 0.67 | 1.685 | 2 | 2 | 0.449 | 0.477 | 4.753 | -27.244 | 66.713 | 134.020 | 1.614 |
| Random | NoComp | PC | 0.67 | 1.685 | 1 | 3 | 0.966 | 0.427 | 2.819 | 41.581 | 60.842 | 121.386 | 1.653 |
| Random | NoComp | PC | 0.67 | 1.685 | 2 | 4 | 0.509 | 0.465 | 1.475 | 21.059 | 54.574 | 215.389 | 8.978 |
| Random | NoComp | PCH | 0.67 | 1.829 | 1 | 1 | 0.647 | 0.477 | 10.980 | -31.459 | 32.945 | 289.634 | 1.119 |
| Random | NoComp | PCH | 0.67 | 1.829 | 2 | 2 | 0.571 | 0.439 | 10.729 | -7.036 | 34.453 | 309.902 | 0.643 |
| Random | NoComp | PCH | 0.67 | 1.829 | 1 | 3 | 0.710 | 0.496 | 9.097 | -176.035 | 40.953 | 205.433 | 1.334 |
| Random | NoComp | PCH | 0.67 | 1.829 | 1 | 4 | 0.691 | 0.465 | 10.710 | -19.573 | 30.039 | 263.225 | 1.070 |
| Random | NoComp | PH | 0.67 | 1.728 | 1 | 1 | 0.370 | 0.427 | 10.384 | -36.753 | 39.417 | 289.336 | 0.542 |
| Random | NoComp | PH | 0.67 | 1.728 | 2 | 2 | 0.399 | 0.427 | 10.623 | -22.810 | 50.076 | 189.982 | 1.275 |
| Random | NoComp | PH | 0.67 | 1.728 | 2 | 3 | 0.417 | 0.477 | 10.648 | 45.482 | 55.964 | 169.967 | 0.582 |
| Random | NoComp | PH | 0.67 | 1.728 | 2 | 4 | 0.773 | 0.443 | 10.309 | -20.245 | 45.128 | 213.397 | 1.123 |
| Random | NoComp | H | 0.67 | 0.144 | 1 | 1 | 0.527 | 0.132 | 9.398 | -2630.081 | 41.876 | 225.801 | 7.050 |
| Random | NoComp | H | 0.67 | 0.144 | 1 | 2 | 0.461 | 0.242 | 11.062 | -7.445 | 41.421 | 157.444 | 0.674 |
| Random | NoComp | H | 0.67 | 0.144 | 1 | 3 | 0.496 | 0.100 | 10.974 | -12.256 | 40.580 | 173.088 | 0.118 |
| Random | NoComp | H | 0.67 | 0.144 | 1 | 4 | 0.753 | 0.163 | 10.541 | 2.078 | 40.691 | 261.225 | 7.403 |
| Random | NoComp | HC | 0.67 | 0.245 | 1 | 1 | 0.816 | 0.308 | 11.093 | 17.542 | 39.576 | 229.678 | 0.422 |
| Random | NoComp | HC | 0.67 | 0.245 | 2 | 2 | 1.333 | 0.276 | 10.478 | 10.244 | 53.603 | 120.772 | 0.989 |
| Random | NoComp | HC | 0.67 | 0.245 | 1 | 3 | 0.433 | 0.314 | 10.867 | 9.282 | 44.287 | 252.188 | 7.132 |
| Random | NoComp | HC | 0.67 | 0.245 | 2 | 4 | 0.654 | 0.295 | 11.087 | 10.772 | 58.091 | 164.772 | 0.370 |
| Rarity | Comp | C | 0.67 | 1.829 | 2 | 1 | 0.596 | 1.174 | 3.152 | -123.948 | 90.375 | 232.877 | 2.157 |
| Rarity | Comp | C | 0.67 | 1.829 | 1 | 2 | 0.928 | 1.365 | 4.847 | -13.828 | 160.564 | 15.446 | 1.509 |
| Rarity | Comp | C | 0.67 | 1.829 | 2 | 3 | 0.654 | 0.954 | 3.271 | -56.320 | 156.395 | 68.941 | 1.629 |
| Rarity | Comp | C | 0.67 | 1.829 | 1 | 4 | 0.791 | 0.891 | 2.241 | -72.204 | 103.736 | 128.818 | 0.996 |
| Rarity | Comp | PC | 0.67 | 1.829 | 3 | 1 | 0.597 | 0.536 | 2.843 | 31.575 | 66.320 | 5.760 | 2.780 |
| Rarity | Comp | PC | 0.67 | 1.829 | 3 | 2 | 0.390 | 0.469 | 2.384 | 5.065 | 57.910 | 281.830 | 2.460 |
| Rarity | Comp | PC | 0.67 | 1.829 | 3 | 3 | 0.643 | 0.473 | 1.692 | 77.383 | 56.270 | 299.690 | 2.830 |
| Rarity | Comp | PC | 0.67 | 1.829 | 3 | 4 | 0.583 | 0.443 | 1.274 | 51.583 | 63.700 | 307.120 | 2.720 |
| Rarity | Comp | PCH | 0.67 | 1.829 | 2 | 1 | 0.349 | 0.439 | 10.867 | 5.104 | 56.687 | 184.019 | 0.893 |
| Rarity | Comp | PCH | 0.67 | 1.829 | 2 | 2 | 0.536 | 0.471 | 10.397 | 3.197 | 62.957 | 133.148 | 0.722 |
| Rarity | Comp | PCH | 0.67 | 1.829 | 1 | 3 | 0.521 | 0.477 | 10.704 | -41.181 | 31.180 | 298.223 | 0.831 |
| Rarity | Comp | PCH | 0.67 | 1.829 | 2 | 4 | 0.565 | 0.414 | 10.679 | -22.288 | 47.414 | 231.648 | 0.849 |
| Rarity | NoComp | C | 0.67 | 0.101 | 2 | 1 | 0.723 | 0.509 | 2.744 | -50.222 | 93.662 | 94.538 | 1.692 |
| Rarity | NoComp | C | 0.67 | 0.101 | 2 | 2 | 0.640 | 0.295 | 2.160 | 29.639 | 57.481 | 151.209 | 7.750 |
| Rarity | NoComp | C | 0.67 | 0.101 | 1 | 3 | 0.854 | 0.308 | 2.536 | -24.474 | 76.534 | 85.565 | 1.441 |
| Rarity | NoComp | C | 0.67 | 0.101 | 1 | 4 | 0.561 | 0.358 | 2.882 | -6.891 | 101.049 | 90.724 | 1.570 |
| Rarity | NoComp | PC | 0.67 | 1.685 | 3 | 3 | 0.467 | 0.455 | 1.716 | 19.754 | 54.090 | 298.830 | 2.660 |
| Rarity | NoComp | PC | 0.67 | 1.685 | 3 | 1 | 0.473 | 0.461 | 1.316 | -1.978 | 63.460 | 268.480 | 2.970 |
| Rarity | NoComp | PC | 0.67 | 1.685 | 3 | 2 | 0.368 | 0.489 | 1.739 | -23.766 | 59.530 | 271.610 | 2.440 |
| Rarity | NoComp | PC | 0.67 | 1.685 | 3 | 4 | 0.299 | 0.440 | 2.047 | 49.420 | 56.820 | 281.290 | 2.310 |
| Rarity | NoComp | PCH | 0.67 | 1.829 | 1 | 1 | 0.552 | 0.433 | 10.962 | -69.001 | 40.290 | 286.543 | 0.959 |
| Rarity | NoComp | PCH | 0.67 | 1.829 | 1 | 2 | 0.598 | 0.496 | 10.911 | 11.490 | 50.147 | 172.692 | 1.016 |
| Rarity | NoComp | PCH | 0.67 | 1.829 | 2 | 3 | 0.679 | 0.427 | 10.554 | -21.035 | 55.523 | 213.651 | 1.277 |
| Rarity | NoComp | PCH | 0.67 | 1.829 | 1 | 4 | 0.446 | 0.414 | 1.400 | -59.946 | 46.079 | 293.506 | 0.891 |
| Bodysize | Comp | P | 1 | 2.000 | 2 | 1 | 0.326 | 0.421 | 0.709 | -20.179 | 56.231 | 218.753 | 1.785 |
| Bodysize | Comp | P | 1 | 2.000 | 2 | 2 | 0.414 | 0.452 | 0.797 | 2.414 | 54.648 | 233.608 | 8.976 |
| Bodysize | Comp | P | 1 | 2.000 | 2 | 3 | 0.289 | 0.433 | 0.766 | 190.905 | 48.687 | 191.521 | 8.159 |
| Bodysize | Comp | P | 1 | 2.000 | 1 | 4 | 0.436 | 0.496 | 1.362 | -4.749 | 73.937 | 103.201 | 2.422 |
| Bodysize | Comp | PC | 1 | 2.000 | 2 | 1 | 0.424 | 0.835 | 2.574 | -8.770 | 114.279 | 77.944 | 2.077 |
| Bodysize | Comp | PC | 1 | 2.000 | 1 | 2 | 0.673 | 0.904 | 3.145 | 0.066 | 59.041 | 177.261 | 1.920 |
| Bodysize | Comp | PC | 1 | 2.000 | 2 | 3 | 0.904 | 0.841 | 2.938 | -22.856 | 51.921 | 245.772 | 1.756 |
| Bodysize | Comp | PC | 1 | 2.000 | 2 | 4 | 0.536 | 1.074 | 3.572 | 31.068 | 90.208 | 98.308 | 1.685 |
| Bodysize | Comp | PCH | 1 | 2.000 | 2 | 1 | 0.835 | 0.804 | 12.067 | -100.495 | 62.242 | 212.827 | 1.255 |
| Bodysize | Comp | PCH | 1 | 2.000 | 2 | 2 | 0.922 | 0.656 | 10.823 | -39.453 | 37.824 | 266.926 | 1.146 |
| Bodysize | Comp | PCH | 1 | 2.000 | 2 | 3 | 0.548 | 0.697 | 11.018 | -61.378 | 83.276 | 165.141 | 0.993 |
| Bodysize | Comp | PCH | 1 | 2.000 | 1 | 4 | 0.760 | 0.760 | 10.936 | 0.717 | 78.657 | 176.780 | 1.272 |
| Bodysize | NoComp | P | 1 | 0.667 | 2 | 1 | 0.527 | 0.358 | 0.898 | 93.293 | 52.081 | 107.595 | 7.630 |
| Bodysize | NoComp | P | 1 | 0.667 | 2 | 2 | 0.523 | 0.396 | 0.898 | -15.220 | 71.755 | 93.573 | 2.273 |
| Bodysize | NoComp | P | 1 | 0.667 | 1 | 3 | 0.383 | 0.402 | 0.804 | 10.929 | 58.903 | 163.016 | 1.005 |
| Bodysize | NoComp | P | 1 | 0.667 | 1 | 4 | 0.540 | 0.389 | 0.753 | -23.208 | 63.225 | 138.611 | 0.129 |
| Bodysize | NoComp | PC | 1 | 1.333 | 1 | 1 | 1.187 | 0.590 | 2.756 | -46.801 | 63.219 | 207.495 | 1.707 |
| Bodysize | NoComp | PC | 1 | 1.333 | 1 | 2 | 0.860 | 0.584 | 2.229 | -30.130 | 66.444 | 168.480 | 1.921 |
| Bodysize | NoComp | PC | 1 | 1.333 | 1 | 3 | 1.331 | 0.703 | 3.039 | -7.371 | 54.913 | 273.524 | 8.661 |
| Bodysize | NoComp | PC | 1 | 1.333 | 2 | 4 | 1.180 | 0.760 | 3.177 | 79.896 | 67.059 | 244.382 | 8.868 |
| Bodysize | NoComp | PCH | 1 | 2.000 | 2 | 1 | 0.380 | 0.929 | 11.884 | -12.511 | 65.176 | 222.376 | 1.499 |
| Bodysize | NoComp | PCH | 1 | 2.000 | 1 | 2 | 0.766 | 0.609 | 10.484 | -16.889 | 36.627 | 391.148 | 8.075 |
| Bodysize | NoComp | PCH | 1 | 2.000 | 2 | 3 | 0.854 | 0.584 | 11.244 | 7.508 | 45.319 | 321.718 | 8.381 |
| Bodysize | NoComp | PCH | 1 | 2.000 | 1 | 4 | 0.891 | 0.722 | 10.830 | -103.050 | 49.982 | 346.495 | 1.768 |
| Random | Comp | C | 1 | 2.000 | 2 | 1 | 0.848 | 1.375 | 3.980 | -65.093 | 105.826 | 209.332 | 1.722 |
| Random | Comp | C | 1 | 2.000 | 2 | 2 | 0.778 | 1.155 | 2.097 | 4.162 | 99.780 | 232.367 | 8.451 |
| Random | Comp | C | 1 | 2.000 | 2 | 3 | 1.122 | 1.300 | 3.221 | -58.079 | 160.716 | 22.450 | 1.350 |
| Random | Comp | C | 1 | 2.000 | 1 | 4 | 0.978 | 1.281 | 5.826 | -33.384 | 172.860 | 60.642 | 1.858 |
| Random | Comp | P | 1 | 2.000 | 1 | 1 | 0.498 | 0.446 | 0.860 | 106.259 | 79.247 | 115.310 | 3.131 |
| Random | Comp | P | 1 | 2.000 | 2 | 2 | 0.245 | 0.421 | 0.735 | 19.437 | 56.472 | 220.651 | 9.290 |
| Random | Comp | P | 1 | 2.000 | 1 | 3 | 0.283 | 0.458 | 0.804 | -49.267 | 57.825 | 196.896 | 1.551 |
| Random | Comp | P | 1 | 2.000 | 2 | 4 | 0.349 | 0.458 | 1.174 | 21.670 | 75.426 | 103.123 | 2.078 |
| Random | Comp | PC | 1 | 2.000 | 2 | 1 | 0.760 | 0.778 | 3.918 | -54.516 | 51.751 | 250.736 | 1.673 |
| Random | Comp | PC | 1 | 2.000 | 2 | 2 | 1.130 | 0.973 | 3.459 | -58.946 | 52.030 | 258.853 | 1.673 |
| Random | Comp | PC | 1 | 2.000 | 2 | 3 | 1.055 | 0.822 | 3.158 | -4.610 | 75.372 | 220.292 | 8.724 |
| Random | Comp | PC | 1 | 2.000 | 2 | 4 | 0.678 | 0.785 | 2.988 | 3.778 | 81.765 | 217.198 | 1.909 |
| Random | Comp | PCH | 1 | 2.000 | 1 | 1 | 0.698 | 0.647 | 11.012 | -11.401 | 52.220 | 236.910 | 1.154 |
| Random | Comp | PCH | 1 | 2.000 | 2 | 2 | 0.791 | 0.860 | 11.200 | -1.654 | 47.521 | 205.767 | 0.872 |
| Random | Comp | PCH | 1 | 2.000 | 2 | 3 | 0.935 | 0.578 | 10.484 | -10.411 | 52.063 | 250.718 | 0.871 |
| Random | Comp | PCH | 1 | 2.000 | 1 | 4 | 0.735 | 0.465 | 10.384 | -39.906 | 63.835 | 307.419 | 1.739 |
| Random | Comp | PH | 1 | 2.000 | 1 | 1 | 0.941 | 0.477 | 10.014 | 0.214 | 24.607 | 236.117 | 0.502 |
| Random | Comp | PH | 1 | 2.000 | 1 | 2 | 0.760 | 0.923 | 10.899 | -55.179 | 13.819 | 196.687 | 0.048 |
| Random | Comp | PH | 1 | 2.000 | 1 | 3 | 0.461 | 0.471 | 10.792 | 6.609 | 16.740 | 281.090 | 0.070 |
| Random | Comp | PH | 1 | 2.000 | 2 | 4 | 0.804 | 0.609 | 10.780 | -16.198 | 22.098 | 343.331 | 0.484 |
| Random | Comp | H | 1 | 2.000 | 1 | 1 | 0.779 | 0.320 | 10.139 | -46.235 | 10.696 | 310.423 | 0.398 |
| Random | Comp | H | 1 | 2.000 | 2 | 2 | 0.542 | 0.565 | 10.773 | -39.144 | 9.111 | 358.766 | 0.168 |
| Random | Comp | H | 1 | 2.000 | 2 | 3 | 0.571 | 0.458 | 10.792 | -69.075 | 5.674 | 434.513 | 0.022 |
| Random | Comp | H | 1 | 2.000 | 1 | 4 | 0.703 | 0.565 | 11.087 | -36.978 | 7.493 | 479.356 | 7.325 |
| Random | Comp | HC | 1 | 2.000 | 1 | 1 | 1.171 | 0.816 | 9.719 | -23.262 | 69.666 | 227.774 | 1.640 |
| Random | Comp | HC | 1 | 2.000 | 2 | 2 | 0.848 | 0.841 | 10.767 | -45.994 | 36.623 | 431.386 | 1.316 |
| Random | Comp | HC | 1 | 2.000 | 1 | 3 | 0.628 | 0.791 | 9.844 | -39.926 | 47.076 | 347.732 | 1.219 |
| Random | Comp | HC | 1 | 2.000 | 2 | 4 | 0.879 | 0.659 | 10.284 | 31.833 | 36.985 | 372.195 | 1.063 |
| Random | NoComp | C | 1 | 0.667 | 1 | 1 | 0.753 | 0.791 | 3.572 | -31.593 | 59.264 | 232.020 | 8.669 |
| Random | NoComp | C | 1 | 0.667 | 1 | 2 | 0.773 | 0.841 | 4.169 | -8.917 | 108.228 | 64.596 | 1.739 |
| Random | NoComp | C | 1 | 0.667 | 1 | 3 | 1.538 | 0.854 | 3.076 | -14.979 | 77.263 | 209.042 | 1.732 |
| Random | NoComp | C | 1 | 0.667 | 2 | 4 | 1.159 | 0.665 | 3.365 | 146.914 | 84.691 | 132.255 | 2.041 |
| Random | NoComp | P | 1 | 0.667 | 1 | 1 | 0.232 | 0.383 | 0.722 | -33.807 | 63.906 | 146.046 | 2.499 |
| Random | NoComp | P | 1 | 0.667 | 2 | 2 | 0.498 | 0.377 | 0.735 | -22.133 | 74.747 | 115.991 | 2.813 |
| Random | NoComp | P | 1 | 0.667 | 1 | 3 | 0.697 | 0.389 | 1.281 | 38.958 | 59.134 | 188.555 | 8.792 |
| Random | NoComp | P | 1 | 0.667 | 2 | 4 | 0.498 | 0.421 | 0.778 | -49.069 | 81.342 | 111.188 | 3.381 |
| Random | NoComp | PC | 1 | 1.333 | 2 | 1 | 0.760 | 0.785 | 3.830 | -38.010 | 81.167 | 99.304 | 1.684 |
| Random | NoComp | PC | 1 | 1.333 | 2 | 2 | 0.665 | 0.728 | 2.436 | 12.182 | 31.666 | 234.674 | 1.570 |
| Random | NoComp | PC | 1 | 1.333 | 2 | 3 | 1.134 | 0.496 | 3.095 | -10.386 | 88.834 | 108.720 | 1.505 |
| Random | NoComp | PC | 1 | 1.333 | 2 | 4 | 0.629 | 0.810 | 3.152 | -12.072 | 115.541 | 70.963 | 1.557 |
| Random | NoComp | PCH | 1 | 2.000 | 1 | 1 | 0.527 | 0.474 | 11.282 | -22.300 | 46.288 | 368.744 | 0.879 |
| Random | NoComp | PCH | 1 | 2.000 | 2 | 2 | 1.171 | 0.866 | 12.600 | -22.650 | 77.503 | 225.690 | 1.967 |
| Random | NoComp | PCH | 1 | 2.000 | 1 | 3 | 0.517 | 0.841 | 10.811 | -48.575 | 77.146 | 190.956 | 1.176 |
| Random | NoComp | PCH | 1 | 2.000 | 1 | 4 | 0.542 | 0.600 | 10.453 | -36.399 | 49.584 | 245.937 | 1.273 |
| Random | NoComp | PH | 1 | 1.333 | 2 | 1 | 0.424 | 0.458 | 11.200 | 2.029 | 24.262 | 268.110 | 0.792 |
| Random | NoComp | PH | 1 | 1.333 | 2 | 2 | 1.078 | 0.584 | 11.238 | -16.389 | 20.352 | 296.204 | 0.473 |
| Random | NoComp | PH | 1 | 1.333 | 1 | 3 | 0.584 | 0.408 | 11.514 | -19.540 | 25.095 | 367.764 | 7.882 |
| Random | NoComp | PH | 1 | 1.333 | 1 | 4 | 0.561 | 0.452 | 10.805 | -19.997 | 21.237 | 285.335 | 0.045 |
| Random | NoComp | H | 1 | 0.667 | 2 | 1 | 0.716 | 0.239 | 10.535 | -0.967 | 17.553 | 395.082 | 7.577 |
| Random | NoComp | H | 1 | 0.667 | 2 | 2 | 0.648 | 0.213 | 11.621 | -40.888 | 30.183 | 214.433 | 0.119 |
| Random | NoComp | H | 1 | 0.667 | 2 | 3 | 0.928 | 0.364 | 10.447 | -123.627 | 22.581 | 252.046 | 0.393 |
| Random | NoComp | H | 1 | 0.667 | 2 | 4 | 0.505 | 0.239 | 11.006 | -4.764 | 32.179 | 205.108 | 0.187 |
| Random | NoComp | HC | 1 | 1.333 | 2 | 1 | 0.985 | 0.684 | 9.668 | -41.683 | 62.577 | 216.447 | 1.111 |
| Random | NoComp | HC | 1 | 1.333 | 2 | 2 | 0.760 | 0.728 | 10.566 | -24.900 | 54.330 | 250.114 | 1.485 |
| Random | NoComp | HC | 1 | 1.333 | 1 | 3 | 1.869 | 0.603 | 11.432 | -90.766 | 49.732 | 241.803 | 1.141 |
| Random | NoComp | HC | 1 | 1.333 | 2 | 4 | 0.778 | 0.483 | 10.629 | 2.647 | 34.913 | 367.210 | 1.768 |
| Rarity | Comp | C | 1 | 2.000 | 4 | 1 | 0.379 | 0.414 | 0.897 | 27.670 | 1.033 | 2.374 | 0.027 |
| Rarity | Comp | C | 1 | 2.000 | 4 | 2 | 0.813 | 0.386 | 0.757 | -9.484 | 0.591 | 1.584 | 0.107 |
| Rarity | Comp | C | 1 | 2.000 | 4 | 3 | 0.967 | 0.372 | 0.918 | -7.785 | 0.659 | 2.270 | 0.045 |
| Rarity | Comp | C | 1 | 2.000 | 4 | 4 | 0.231 | 0.372 | 0.946 | -4.100 | 1.029 | 2.766 | 0.041 |
| Rarity | Comp | PC | 1 | 2.000 | 4 | 1 | 0.862 | 1.122 | 3.000 | -31.599 | 1.480 | 2.109 | 0.034 |
| Rarity | Comp | PC | 1 | 2.000 | 4 | 2 | 0.803 | 1.255 | 3.197 | -32.156 | 0.938 | 2.437 | -0.005 |
| Rarity | Comp | PC | 1 | 2.000 | 4 | 3 | 1.164 | 0.796 | 2.734 | -20.467 | 1.283 | 2.929 | 0.006 |
| Rarity | Comp | PC | 1 | 2.000 | 4 | 4 | 0.806 | 1.178 | 2.447 | 1.579 | 1.135 | 1.617 | 0.044 |
| Rarity | Comp | PCH | 1 | 2.000 | 4 | 1 | 1.040 | 1.002 | 10.600 | -0.282 | 0.636 | 2.782 | 0.000 |
| Rarity | Comp | PCH | 1 | 2.000 | 4 | 2 | 1.290 | 1.178 | 10.354 | -57.262 | 0.514 | 3.864 | -0.011 |
| Rarity | Comp | PCH | 1 | 2.000 | 4 | 3 | 1.104 | 0.778 | 9.836 | 5.457 | 0.897 | 3.600 | 0.036 |
| Rarity | Comp | PCH | 1 | 2.000 | 4 | 4 | 1.024 | 1.157 | 11.111 | -62.060 | 0.444 | 4.495 | 0.006 |
| Rarity | NoComp | C | 1 | 0.667 | 2 | 1 | 0.923 | 0.697 | 3.447 | 8.977 | 51.853 | 254.335 | 8.492 |
| Rarity | NoComp | C | 1 | 0.667 | 1 | 2 | 0.897 | 0.778 | 3.064 | 3.031 | 111.000 | 75.085 | 1.656 |
| Rarity | NoComp | C | 1 | 0.667 | 1 | 3 | 0.723 | 0.747 | 3.509 | 8.085 | 102.675 | 83.901 | 1.848 |
| Rarity | NoComp | C | 1 | 0.667 | 1 | 4 | 0.897 | 0.935 | 3.892 | -112.971 | 97.545 | 82.815 | 1.475 |
| Rarity | NoComp | PC | 1 | 1.333 | 1 | 1 | 0.735 | 0.709 | 2.907 | 29.258 | 61.463 | 232.306 | 9.032 |
| Rarity | NoComp | PC | 1 | 1.333 | 2 | 2 | 0.617 | 0.722 | 2.574 | -34.644 | 90.401 | 98.800 | 1.579 |
| Rarity | NoComp | PC | 1 | 1.333 | 2 | 3 | 0.904 | 0.747 | 3.064 | 21.677 | 46.721 | 282.808 | 9.205 |
| Rarity | NoComp | PC | 1 | 1.333 | 2 | 4 | 0.673 | 0.791 | 5.619 | 3.878 | 89.868 | 62.273 | 1.443 |
| Rarity | NoComp | PCH | 1 | 2.000 | 1 | 1 | 0.810 | 0.684 | 11.075 | -13.062 | 44.231 | 449.084 | 8.909 |
| Rarity | NoComp | PCH | 1 | 2.000 | 1 | 2 | 1.161 | 0.515 | 10.729 | -62.543 | 38.766 | 334.588 | 1.516 |
| Rarity | NoComp | PCH | 1 | 2.000 | 2 | 3 | 1.034 | 0.785 | 11.100 | -14.187 | 62.356 | 246.033 | 1.572 |
| Rarity | NoComp | PCH | 1 | 2.000 | 2 | 4 | 0.659 | 0.665 | 10.623 | -31.519 | 34.330 | 364.686 | 1.372 |
